# Epigallocatechin-3-gallate chitosan nanoparticles in an extender improve the antioxidant capacity and post-thawed quality of Kacang goat semen

**DOI:** 10.12688/f1000research.127744.1

**Published:** 2023-01-09

**Authors:** Imam Mustofa, Suherni Susilowati, Tri Wahyu Suprayogi, Adeyinka Oye Akintunde, Yudit Oktanella, Djoko Agus Purwanto

**Affiliations:** 1 Division of Veterinary Reproduction, Faculty of Veterinary Medicine, Airlangga University, Surabaya, East Java, 60115, Indonesia; 2 Department of Agriculture and Industrial Technology, Babcock University, Ilishan-Remo, Ogun State, Nigeria; 3 Department of Veterinary Reproduction, Faculty of Veterinary Medicine, Brawijaya University, Malang, East Java, Indonesia; 4 Department of Pharmaceutical Chemistry, Faculty of Pharmacy, Airlangga University, Surabaya, East Java, 60115, Indonesia

**Keywords:** catalase, extinction risk, radical scavenging, smallholder and farm, sperm motility

## Abstract

**Background and Aim:** The Kacang goat ( *Capra hircus*) is an indigenous livestock species in Indonesia that is at risk of extinction due to cross-breeding. Artificial insemination (AI) techniques are expected to increase the population of these goats. This study aimed to determine the addition of epigallocatechin-3-gallate chitosan nanoparticles (EGCG CNPs) to skim milk–egg yolk (SM–EY) extender to obtain the best possible quality of post-thawed Kacang buck semen for AI.

**Materials and Methods:** Fresh Kacang buck semen was diluted in SM–EY without or with the addition of 0.5, 1.0, 1.5, or 2.0 µg of EGCG CNPs/mL extender. Extended semen was packaged in French mini straws, frooze, and stored in liquid nitrogen at −196℃ for 24 hours. Six replicates from each treatment group were thawed for catalase, 2,2-diphenyl-1-picrylhydrazyl (DPPH) radical scavenging, malondialdehyde (MDA), sperm intact plasma membrane (IPM), viability and motility analyses.

**Results:** Post-thawed semen that was previously frozen without EGCG CNPs in the extender (control group) exhibited the lowest levels of catalase, DPPH, sperm viability, sperm motility, IPM, and the highest levels of MDA. However, the addition of EGCG CNPs at doses of 1.5 µg/mL extender increased post-thawed catalase, DPPH, sperm IPM, viability, and sperm motility and decreased MDA levels (p < 0.05) than those of control group.

**Conclusion:** This study was the first in which EGCG CNPs were used in SM–EY extender, and the addition of only 1.0 µg/mL of EGCG CNPs in this extender increased the antioxidant capacity and post-thawed quality of Kacang buck semen.

## Introduction

Smallholder farmers raise Kacang goats ( *Capra hircus*) to increase their financial income, reduce poverty, and prevent malnutrition. However, the pure breed of this indigenous livestock species in Indonesia is at risk of extinction due to cross-breeding and must be protected. ^1^ Artificial insemination (AI) techniques involving freeze–thawed semen are expected to increase the population of these goats. However, goat sperm is sensitive to cold shock. ^2^ Indeed, >60% sperm death was detected in post-thawed goat semen previously frozen in skim milk–egg yolk (SM–EY) extender without antioxidants, ^3^ which does not meet the minimum requirements for AI, i.e., motility must be >40%. ^4^ High sperm death and low motility occurs because the freezing and thawing process leads to an excess of reactive oxygen species (ROS) production, which damages the polyunsaturated fatty acids (PUFAs) in the plasma membrane of spermatozoa, increasing malondialdehyde (MDA) levels and ultimately reducing sperm viability and motility. ^5^ Therefore, antioxidants are needed to counteract the effects of ROS and thereby increase post-thawed semen quality. ^6^ In our previous studies, green tea extract was used to improve semen quality and decrease nucleotide mutations in mtDNA ^3^ and protein-encoded mtDNA, ^7^ presumably due to an increase in the antioxidant capacity.

The freeze–thawing process in goat semen causes lipid peroxidation in the spermatozoa membrane, which reduces semen quality. ^8^ Semen contains endogenous antioxidants that help maintain the oxidant–antioxidant balance. ^9^ However, the excessive production of ROS due to the freeze–thawing process cannot be overcome by endogenous antioxidants owing to the limitations of the spermatozoa cytoplasm and decreased antioxidant levels due to the addition of extenders. ^10^ Epigallocatechin-3-gallate (EGCG) is a powerful antioxidant extracted from green tea. ^11^ The nanoparticle extract has a large surface area to volume ratio, so it is expected to exhibit increased penetration into cells, ^12^ including sperm cells, and improve the quality of post-thawed semen. The addition of EGCG chitosan nanoparticles (CNPs) to the extender when freezing Kacang goat semen has not yet been studied. Thus, in the present study, the addition of EGCG CNPs to SM–EY extender and their ability to increase antioxidant capacity was investigated by assessing catalase and 2,2-diphenyl-1-picrylhydrazyl (DPPH) levels. The aim of this study was to obtain the best possible quality of post-thawed Kacang buck semen for AI according to MDA levels, sperm intact plasma membrane (IPM), viability, and motility.

## Methods

The study was conducted in February to August 2022, at The Artificial Insemination Center of Airlangga University, Tanjung village, Kedamean, Gresik District, East Java, Indonesia, at coordinates 7° 19′ 25′′ S and 112° 32′ 54′′ E.

### Ethical approval

This study is part of a multiyear research project. The protocol was approved by the Animal Care and Use Committee of Airlangga University (number 520/HRECC.FODM/VII/2019).

### Preparation of EGCG CNPs

Dried green tea ( *Camellia sinensis.* Kuntze) leaves was obtained from Perkebunan Nusantara XII Malang, East Jawa Indonesia. Briefly, EGCG was isolated from *C. sinensis* using a thin-layer chromatography method and verified by a comparison with epigallocatechin gallate hydrate (Tokyo Chemical Co., Ltd., Japan). ^13^ Subsequently, 5 mL of 0.1% chitosan solution [containing low molecular weight chitosan (Sigma-Aldrich) in 1% acetic acid] was added to 50 mL of EGCG solution [0.05% EGCG (Sigma-Aldrich) in distilled water], and the mixture was stirred at room temperature. Next, 0.5 mL of triphosphate (TPP) solution [0.025% TPP (Merck) in distilled water] was added drop-wise with stirring for 3 h at 112 × *g.* This solution was centrifuged at 21,952 × *g* for 10 min using a MC-10K centrifuge (Bio-Gener, Hangzhou, China) and washed three times with deionized water to obtain the EGCG CNPs, which were freeze dried for 48 h and stored at 4°C. ^14^ The particle size of EGCG CNPs was measured using a Zetasizer Nano ZS (ZEN 3600, Malvern Instruments Ltd., Worcestershire, UK). A helium–neon ion laser at a wavelength of 633 nm was used as the incident beam at 25°C with a 90° angle. ^15^

### Experimental animals

Samples were collected from three of Kacang bucks aged two–three years and weighing 35–40 kg. These bucks were (owned by The Artificial Insemination Center, Airlangga University) fed approximately four kg of forage and 3.5 kg of concentrate (16%–18% crude protein) daily and provided with drinking water *ad libitum.* Semen was collected from the bucks using an artificial vagina twice per week to obtain six ejaculate samples to process as frozen semen.

### SM–EY extender

Skim milk powder (15 g; 115338; Merck) was dissolved in distilled water to a volume of 150 mL, heated for 10 min to 92°C–95°C, and then cooled to room temperature (25°C). Egg yolk (5 mL; derived from laboratory chicken eggs) was added to 95 mL of skim milk solution, then added with 1 IU/mL of penicillin (Meiji Seika Pharma, Tokyo, Japan) and 1 μg/mL of streptomycin (Thermo Fisher Scientific, Singapore). ^7^ The solution was divided into five equal volumes without addition of EGCG CNPs for the control group (T0) and with addition of 0.5, 1.0, 1.5, and 2.0 μg of EGCG CNPs/mL extender for T1, T2, T3 and T4 groups, respectively.

### Frozen semen

Each SM–EY extender group was divided into two equal volumes. The first volume was added to fresh semen to obtain 480 million spermatozoa/mL. The second volume was added with glycerol up to 16% concentration, which was in turn added to the first mixture to obtain 240 million spermatozoa/mL. The extended semen was cooled from room temperature (25°C) to 5°C for 1 hour, then filled in 0.25 ml French straws (I.M.V., France) and sealed. The filled and sealed straws were chilled in liquid nitrogen vapor from 5°C to − 140°C for 10 min, and immediately stored in liquid nitrogen (−196°C) for 24 hours before evaluation were conducted. ^7^

### Evaluation of post-thawed sperm quality

The straws allocated to each group were thawed in sterile water for 30 s at 37°C. Six replicates randomly were used to assess sperm IPM, viability, progressive motility, MDA levels, catalase levels, and DPPH scavenging, respectively, according to methods reported in a previous study. ^7^

***IPM***

A semen sample (0.1 mL) was added to 1 mL of a hypoosmotic solution [containing 7.35 g of sodium citrate (Sigma-Aldrich) and 13.52 g of fructose (Sigma-Aldrich) dissolved in distilled water to a volume of 1 L)] and incubated at 37°C for 30 min. The sperm IPM was assessed for 100 sperm under a light microscope (Olympus BX-53, Tokyo, Japan) at 400× magnification. Sperm with an IPM showed a curved tail, whereas those with a damaged plasma membrane showed a straight tail. ^7^

***Viability***

A drop of semen sample and a drop of nigrosine (Sigma-Aldrich) were mixed and smeared on a glass slide, after which the slide was dried over a flame. The slide was then examined under a light microscope (Olympus BX-53, Tokyo, Japan) at 400× magnification to evaluate the percentage of live sperm in 100 spermatozoa. Live sperm were identified by their brightly transparent heads, whereas dead sperm were colored red. ^7^

***Motility***

An homogenate of a semen sample (10 μL) and a 0.9% (w/v) NaCl solution (1 mL) was dropped onto a glass slide and covered. The number of progressively motile sperm was counted for 100 sperm at 400× magnification under a light microscope (Olympus BX-53, Tokyo, Japan) equipped with Linkam Warming Stages set at 37°C–38°C (Meyer Instruments, Texas, USA). ^7^

***MDA levels***

MDA levels in semen samples were determined using the thiobarbituric acid (Sigma-Aldrich) method. A semen sample (100 μL) and MDA kits (0, 1, 2, 3, 4, 5, 6, 7, and 8 μg/mL of malondialdehyde) were added to 550 μL of distilled water and 100 μL of 20% trichloroacetic acid. These mixtures were then homogenized for 30 s, and 250 μL of HCl (1 N) was then added and homogenized. Subsequently, 100 μL of 1% sodium thiobarbiturate was added and homogenized. This mixture was centrifuged at 28 × *g* for 10 min, and the supernatant was incubated in a 100°C water bath for 30 min before being left to room temperature (25°C). The color absorption was determined at a wavelength of 533 nm using a spectrophotometer (Thermo Fisher Scientific). MDA levels (ng/mL) were determined by extrapolating the sample absorbance values using a standard MDA curve. ^7^

***Catalase levels***

A 30 mM H _2_O _2_ phosphate-buffered solution [1 mL; comprising 0.34 mL of 30% H _2_O _2_ diluted in fresh phosphate buffer (50 mM and pH 7)] was added to a semen sample (2 mL) at room temperature and assessed against a blank (not containing an enzyme) solution. The UV spectrophotometric absorbance method was used to measure catalase activity at a wavelength of 240 nm. ^16^

***DPPH radical scavenging***

A 5 mL of DPPH radicals (10 mM) in methanol were added to a cuvette containing 970 mL of mixed methanol. This mixture was incubated at 20°C for 3 min, and the absorbance was measured at 517 nm (A517) using a UV-Vis Spectrophotometer (Thermo Fisher Scientific). Next, 25 mL of each sample and 25 mL of an acetonitrile solution (9.5 M; used as negative control) were added and mixed, and the mixture was incubated at 20°C for 3 min. Subsequently, the A517 decrease related to DPPH radical decomposition was measured. All experiments were performed in duplicate, and the mean DPPH scavenging effect was calculated according to the following formula: DPPH scavenging effect (%) = (1 − A517 sample/A517 negative control) × 100. ^17^ IC _50_ values were calculated using a relationship curve of RSA versus concentrations of the respective sample curve. ^18^

### Data analysis

ANOVA followed by the Tukey Honestly Significant Difference test were used to analyze these data with a significance level of p ≤ 0.05. SPSS (Version 23, IBM Corp., Armonk, NY, USA) was used for data analysis.

## Results

**Figure 1.**
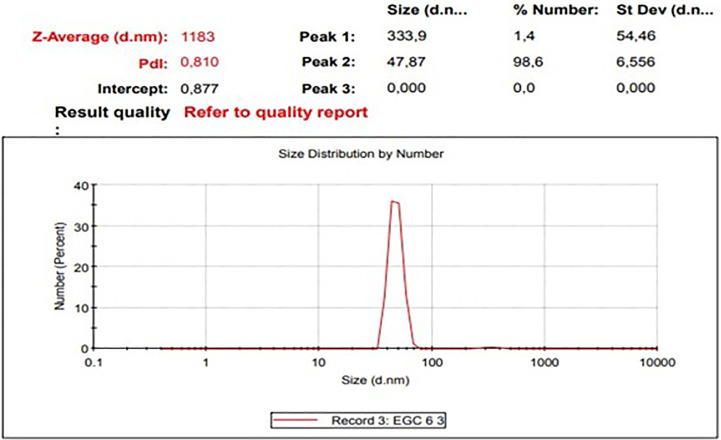
Size distribution curves of EGCG CNPs (Zetasizer Ver. 7.01 (MAL1061025, Malvern Instruments Ltd, Worcs., UK)).

The diameters particles of EGCG CNPs was in range 41.31 – 388.36 nm with the averages as presented in Table 1 and size distribution curves of EGCG CNPs as seen in Figure 1. The progressive motility of sperm indicates the quality of fresh semen. Based on the criteria for the motility of individual spermatozoa of >70%, the obtained semen ejaculate of the Kacang goats met the requirements for freezing ( Table 2).

**Table 1.** Size (nm) and distribution of EGCG CNPs.

Range	Averages	Percentage
10–100	47.87 ± 6.56	98.6%
100–1000	333.9 ± 54.46	1.4%

**Table 2.** Macroscopic and microscopic parameters of fresh Kacang buck semen.

Parameter	Value
Volume (mL)	1.37 ± 0.06
Color	Creamy white (normal)
pH	6–7
Consistency	Thick (normal)
Concentration (million/mL)	1933.67 ± 51.03
Mass motility	+++ (normal)
Progressive motility (%)	83.33 ± 2.89
Viability (%)	88.33 ± 1.52

Post-thawed semen that was previously frozen without antioxidant EGCG CNPs in the extender (T0 group) exhibited the lowest levels of catalase and DPPH and the highest levels of MDA (p < 0.05). The addition of EGCG CNPs in extender resulted in higher catalase and DPPH levels and lower MDA levels (p < 0.05) in optimum dose of 1.5 μg/mL of extender (T3) than those in the T0 group (p < 0.05) ( Table 3).

**Table 3.** Post-thawed sperm malondialdehyde (MDA; nmol/mL), catalase (× 10 ^−3^ U/mg), and 2,2-diphenyl-1-picrylhydrazyl (DPPH; %) levels in Kacang buck semen extended in skim milk–egg yolk (SM–EY) with or without the addition of epigallocatechin-3-gallate nanoparticles (EGCG CNPs).

Groups	Catalase	DPPH	MDA
T0	43.07 ± 1.75 ^a^	68.37 ± 3.88 ^a^	2096.28 ± 45.16 ^b^
T1	50.28 ± 1.87 ^b^	74.55 ± 3.29 ^a^	1779.51 ± 55.10 ^b^
T2	61.40 ± 2.44 ^c^	82.16 ± 1.35 ^b^	1381.17 ± 65.70 ^a^
T3	67.68 ± 2.36 ^d^	86.04 ± 1.28 ^c^	1250.22 ± 43.06 ^a^
T4	69.62 ± 2.82 ^d^	87.39 ± 1.48 ^c^	1356.74 ± 62.41 ^a^

T0: Kacang buck semen in SM–EY without EGCG CNPs; T1, T2, T3: Kacang buck semen in SM–EY added with 0.5, 1.0, 1.5, 2.0 μg of EGCG CNPs/mL. Different superscript in the same column show significant differences (p < 0.05).

Semen that was frozen without antioxidant EGCG CNPs in the extender (T0 group) exhibited lowest sperm IPM, viability, and motility both in the pre-freezing and post-thawed conditions (p < 0.05). The addition of EGCG CNPs at 1.5 μg/mL of extender (T3) was optimum dose for increasing of sperm viability, motility and IPM (p < 0.05) than those in the T0 group ( Table 4).

**Table 4.** Pre-freezing and post-thawed sperm viability, progressive motility, and intact plasma membrane (IPM) of Kacang buck semen extended in skim milk–egg yolk (SM–EY) with or without the addition of epigallocatechin-3-gallate nanoparticles (EGCG CNPs).

Groups	IPM		Viability		Motility	
	Pre-freezing	Post-thawed	Pre-freezing	Post-thawed	Pre-freezing	Post-thawed
T0	47.17 ± 2.56 ^a^	30.33 ± 3.08 ^a^	58.33 ± 2.58 ^a^	33.33 ± 2.66 ^a^	54.33 ± 3.50 ^a^	30.00 ± 4.47 ^a^
T1	53.50 ± 4.85 ^a^	31.17 ± 3.06 ^a^	60.83 ± 3.76 ^a^	36.67 ± 2.58 ^a^	57.83 ± 4.58 ^a^	33.50 ± 3.21 ^a^
T2	57.67 ± 3.50 ^a^	34.33 ± 2.25 ^a^	65.00 ± 3.16 ^ab^	40.83 ± 2.04 ^ab^	61.67 ± 3.61 ^ab^	38.17 ± 2.40 ^ab^
T3	65.17 ± 5.64 ^ab^	38.00 ± 2.61 ^ab^	72.50 ± 2.74 ^b^	42.50 ± 2.74 ^b^	67.83 ± 3.43 ^b^	40.33 ± 2.25 ^b^
T4	64.67 ± 4.37 ^ab^	40.17 ± 4.67 ^b^	75.00 ± 4.47 ^b^	44.17 ± 2.04 ^b^	71.83 ± 4.02 ^b^	43.50 ± 4.81 ^b^

T0: Kacang buck semen in SM–EY without EGCG CNPs; T1, T2, T3: Kacang buck semen in SM–EY added with 0.5, 1.0, 1.5, 2.0 μg of EGCG CNPs/mL. Different superscript in the same column show significant differences (p < 0.05).

## Discussion

Nanotechnology techniques have been used to produce particles with a size scale of 0.1–1000 nm. ^19^ The smaller particle size of nanoparticles than the microparticles, causes the NPs have larger surface areas to volume ratio and the opportunities for chemical reactions and biological activities also increase. Bioavailability is the ability of NPs to penetrate cells. The effect of NPs on the target site depends on their chemical composition, shape, surface structure, surface charge, catalytic properties, and aggregation ability with other materials. ^20^ The NPs size causes the active compound to spread in the medium and reach the target with increased accuracy. ^21^ One of the safest materials used in NP encapsulation technology is chitosan. ^22^

EGCG possesses metal-chelating properties that provide antioxidant functions. The two structures that give EGCGs metal chelation properties are the ortho-3′,4′-dihydroxy moiety and the 4-keto, 3-hydroxyl, or 4-keto and 5-hydroxyl moiety. Catechins prevent the generation of potentially damaging free radicals via the chelation of metal ions. Through their ability to chelate transition metal ions, flavonoids can complex and inactivate iron ions, thereby suppressing the superoxide-driven Fenton reactions that are thought to be a crucial route to forming ROS. Electron transfer from catechins to ROS-induced radical sites on DNA and the formation of stable semiquinone free radicals are other mechanisms by which catechins exert their antioxidant effects, ^23^ which are more pronounced than those of vitamins C and E. ^24^

### Antioxidant capacity

Semen has antioxidant enzymes, namely catalase, superoxide dismutase (SOD), and glutathione peroxidase (GPX), which maintain the oxidant–antioxidant balance ^25^ and play fundamental roles in protecting biological systems against free radical attacks. The scavenging activity of SOD is accomplished via catalase, which reduces hydrogen peroxide to water and molecular oxygen. ^26^ Indeed, catalase activates the decomposition of hydrogen peroxide into water and oxygen, thereby blocking the ROS-generating pathway and reducing oxidative stress. ^27^ The addition of catalase to a commercially medium increased the total motility, membrane integrity, and viability of postliquid goat semen ^28^ and ram semen. ^29^ Optimal catalase levels in the extender also reduce detrimental effects on post-thawed motility, viability, plasma membrane, and acrosome integrity. ^30^ In humans, catalase is used as a molecular target for diagnosing and monitoring male infertility ^31^ and in strategies for optimizing sperm parameters. ^32^ This study’s result is consistent with that of Papas *et al.,* ^33^ who demonstrated that the specific activities of catalase, GPX, and glutathione reductase during stallion semen cryopreservation were similar between effective and ineffective freezing of ejaculates. However, SOD activity was found to be higher in ejaculate following effective freezing than in ejaculate subjected to poor freezing power. ^34^ In stallions, the total and specific activity of catalase in seminal plasma is high; however, no correlation was observed between total catalase activity in stallion seminal plasma and sperm kinematic parameters. ^33^

A higher value of DPPH in the T3 than those of T0 group represents more effective free radical scavenging. ^35^ A DPPH measurement has an acceptable reproducibility for determination of radical scavenging activity in several samples. ^36^ The DPPH assay, a popular method for evaluating the kinetics and stoichiometry of antioxidative reactions, is used widely because it is easy to use, rapid, and sensitive. The assay is based on the reduction of the purple chromogen DPPH· via a hydrogen atom or electron transfer from the scavenging molecule, i.e., an antioxidant, which causes the formation of pale yellow hydrazine (i.e., DPPH). ^37^

The highest levels of MDA in the control group indicate highest lipid peroxidation. Oxidative stress may result in an imbalance between ROS generation and endogenous antioxidant activities. Higher ROS levels cause cell damage through the peroxidation of PUFAs in the sperm plasma membrane. Lipid peroxidation generates toxic lipid aldehyde species, including MDA, ^38^ and higher MDA levels indicate free radical attacks ^39^ and plasma membrane damage. ^40^ The lower MDA levels in post-tahwed semen of T3 group indicated the decreasing of oxidative stress, followed by an increase in membrane fluidity and a lower percentage of acrosomal damage owing to the rearrangement of membrane lipids and proteins. ^27^

### Quality of post-thawed semen

Goat semen is sensitive to cryopreservation. Freezing semen leads to excessive ROS production ^41^ followed by lipid peroxidation of the membrane, resulting in MDA production, ^42^ which in turn markedly reduces sperm IPM, viability, motility, and DNA integrity. ^43^

Intactness of plasma membrane is essential for protecting the organelles of sperm and molecular transportation; thus, it is crucial for sperm viability and sperm motility. ^44^ Post-thawed semen previously frozen without antioxidant EGCG CNPs in the extender showed the lowest IPM, sperm viability, and sperm motility levels, whereas adding EGCG CNPs to the extender increased each of these levels. Damage to the plasma membrane of the spermatozoa reduces the quality of post-thawed semen because the integrity of this membrane is essential for the survival and motility of sperm. ^45^ Excessive ROS production in semen due to the freezing–thawing process causes lipid peroxidation in the sperm membrane, ^8^ disrupting the structure and function of this membrane and leading to the death of the spermatozoa. ^43^ Lipid peroxidation also damages axonemal and mitochondrial proteins, resulting in the loss of sperm motility, even though the spermatozoa remain alive. ^46^ Membrane damage due to lipid peroxidation also results in higher MDA levels. ^47^ Furthermore, ROS cause the opening of bonds between disulfide chains in proteins, thereby destabilizing the DNA structure and leading to DNA fragmentation. ^48^ The ejaculate defense system, including antioxidant enzymes such as GPX, catalase, and SOD, deals with ROS. ^49^ However, the smaller volume of the sperm cytoplasm than those of commonly cells is the challenging for the antioxidant. ^50^ The addition of semen extender might also lead to decreases of endogenous antioxidants. Insufficient antioxidant levels to combat oxidative stress during cryopreservation can have multiple negative effects, including decreased sperm viability, sperm motility, and plasma sperm integrity. Thus, the addition of antioxidants to the extender prior to semen freezing is necessary.

The EGCG is an antioxidant that can reduce lipid peroxidation, protein carbonylation, and sperm DNA damage. ^51^ The NPs form of EGCG increases the ratio of surface area of membrane to volume of particles, which allows EGCG to penetrate sperm cells efficiently. ^52^ The presence of antioxidants reduces lipid peroxidation in the plasma membrane of spermatozoa, which in turn increases sperm viability, motility, and acrosome integrity. ^53^ Adding EGCG CNPs at 1.5 μg/mL of SM–EY extender improved semen quality compared with that of the control group. These results are consistent with those of previous studies in which adding ethanol green tea extract in extender maintained the motility, viability, IPM, and DNA integrity of Simmental bull sperm. ^54^

## Conclusion

In this study, EGCG CNPs were used for the first time in SM–EY extender to improve the antioxidant capacity and quality of post-thawed semen. Indeed, the addition of 1.5 μg/mL EGCG CNPs to the SM–EY extender increased the antioxidant capacity and quality of Kacang buck post-thawed semen. Future studies are required to validate this finding for AI under farm conditions.

## Author’s contributions

Imam Mustofa (IM), Suherni Susilowat I (SS), Tri Wahyu Suparyogi (TWS), Adeyinka Oye Akintunde (AOA), Yudit Oktanella (YO), Djoko Agus Purwanto (DAP)

IM, SS and TWS conceived the idea, designed the mainframe of this study, and conceived in detail the manuscript. IM, TWS collected, prepared extender and freezing semen process. DAP: extracted EGCG from green tea leaves. YO: processing EGCG nanoparticles and measuring the particle size of extenders. SS, YO: evaluated semen quality. IM: interpreted the data and statistical analysi. AOA, YO, DAP: read critically and revised the manuscript for intellectual content. All authors read and approved the final manuscript.

## Data Availability

Biostudies. Epigallocatechin-3-gallate chitosan nanoparticles in an extender improve the antioxidant capacity and post-thawed quality of Kacang Goat semen. DOI: https://www.ebi.ac.uk/biostudies/studies/S-BSST924 ^55^ This project contains the following data:
- This study aimed to determine the addition of epigallocatechin-3-gallate chitosan nanoparticles (EGCG CNPs) to skim milk–egg yolk (SM–EY) extender to obtain the best possible quality of post-thawed Kacang buck semen for AI ARRIVE checklist. Figshare. DOI: https://doi.org/10.6084/m9.figshare.21531213.v1 ^56^

## References

[1] Suswono S Decree of the Minister of Agriculture of the Republic of Indonesia Number 2840/Kits/LB.430/8/2012. 2012. Accessed June 17 2022. Reference Source

[2] Hahn K Failing K Wehrend A : Effect of temperature and time after collection on buck sperm quality. *BMC Vet. Res.* 2019;15:355. 10.1186/s12917-019-2135-y 31640772 PMC6823624

[3] Mustofa I Susilowati S Wurlina W : Green tea extract increases the quality and reduced DNA mutation of post-thawed Kacang buck sperm. *Heliyon.* 2021;7:e06372. 10.1016/j.heliyon.2021.e06372 33732926 PMC7944040

[4] INSA (Indonesian National Standard Agency): Frozen semen–Part 3: goat and sheep. Jakarta; Retrieved June 17 2019. 2014. Reference Source

[5] Al-Mutary MG : Use of antioxidants to augment semen efficiency during liquid storage and cryopreservation in livestock animals: a review. *J. King Saud Univ. Sci.* 2021;33:101226–101226. 10.1016/j.jksus.2020.10.023

[6] Arando Arbulu A Navas González FJ Bermúdez-Oria A : Bayesian Analysis of the Effects of Olive Oil-Derived Antioxidants on Cryopreserved Buck Sperm Parameters. *Animals (Basel).* 2021;11:2032. 10.3390/ani11072032 34359159 PMC8300210

[7] Susilowati S Mustofa I Wurlina W : Green Tea Extract in the Extender Improved the Post-Thawed Semen Quality and Decreased Amino Acid Mutation of Kacang Buck Sperm. *Vet. Sci.* 2022;9:403. 10.3390/vetsci9080403 36006318 PMC9413626

[8] Sabeti P Pourmasumi S Rahiminia T : Etiologies of sperm oxidative stress. *Int. J. Reprod. Biomed.* 2016;14:231–240. 10.29252/ijrm.14.4.231 27351024 PMC4918773

[9] Kowalczyk A : The Role of the Natural Antioxidant Mechanism in Sperm Cells. *Reprod. Sci.* 2022;29:1387–1394. 10.1007/s43032-021-00795-w 34845666 PMC9005387

[10] Riesco MF Alvarez M Anel-Lopez L : Multiparametric Study of Antioxidant Effect on Ram Sperm Cryopreservation-From Field Trials to Research Bench. *Animals (Basel).* 2021;11:283. 10.3390/ani11020283 33498656 PMC7911426

[11] Chu C Deng J Man Y : Green Tea Extracts Epigallocatechin-3-gallate for Different Treatments. *Biomed. Res. Int.* 2017;2017:5615647. 28884125 10.1155/2017/5615647 PMC5572593

[12] Mustapha T Misni N Ithnin NR : A Review on Plants and Microorganisms Mediated Synthesis of Silver Nanoparticles, Role of Plants Metabolites and Applications. *Int. J. Environ. Res. Public Health.* 2022;19:674. 10.3390/ijerph19020674 35055505 PMC8775445

[13] Zhao W Liu Z Liang X : Preparation and characterization of epigallocatechin-3-gallate loaded melanin nanocomposite (EGCG @MNPs) for improved thermal stability, antioxidant and antibacterial activity. *LWT Food Sci. Technol.* 2022;154:112599. 10.1016/j.lwt.2021.112599

[14] Safer AM Leporatti S Jose J : Conjugation Of EGCG And Chitosan NPs As A Novel Nano-Drug Delivery System. *Int. J. Nanomedicine.* 2019;14:8033–8046. 10.2147/IJN.S217898 31632016 PMC6781949

[15] Nguyen TL Nguyen TH Nguyen DH : Development and *In vitro* Evaluation of Liposomes Using Soy Lecithin to Encapsulate Paclitaxel. *Int. J. Biomater.* 2017;2017:1–7. 10.1155/2017/8234712 PMC5346369 28331495

[16] Hadwan MH : Simple spectrophotometric assay for measuring catalase activity in biological tissues. *BMC Biochem.* 2018;19:7. 10.1186/s12858-018-0097-5 30075706 PMC6091033

[17] Chrzczanowicz J Gawron A Zwolinska A : Simple method for determining human serum 2,2-diphenyl-1-picryl-hydrazyl (DPPH) radical scavenging activity - possible application in clinical studies on dietary antioxidants. *Clin. Chem. Lab. Med.* 2008;46:342–349. 10.1515/CCLM.2008.062 18254708

[18] Rivero-Cruz JF Granados-Pineda J Pedraza-Chaverri J : Phytochemical Constituents, Antioxidant, Cytotoxic, and Antimicrobial Activities of the Ethanolic Extract of Mexican Brown Propolis. *Antioxidants (Basel).* 2020;9:70. 10.3390/antiox9010070 31940981 PMC7022611

[19] Danaei M Dehghankhold M Ataei S : Impact of Particle Size and Polydispersity Index on the Clinical Applications of Lipidic Nanocarrier Systems. *Pharmaceutics.* 2018;10:57. 10.3390/pharmaceutics10020057 29783687 PMC6027495

[20] Jeevanandam J Barhoum A Chan YS : Review on nanoparticles and nanostructured materials: history, sources, toxicity and regulations. *Beilstein J. Nanotechnol.* 2018;9:1050–1074. 10.3762/bjnano.9.98 29719757 PMC5905289

[21] Mitchell MJ Billingsley MM Haley RM : Engineering precision nanoparticles for drug delivery. *Nat. Rev. Drug Discov.* 2021;20:101–124. 10.1038/s41573-020-0090-8 33277608 PMC7717100

[22] Mohammed MA Syeda JTM Wasan KM : An Overview of Chitosan Nanoparticles and Its Application in Non-Parenteral Drug Delivery. *Pharmaceutics.* 2017;9:53. 10.3390/pharmaceutics9040053 29156634 PMC5750659

[23] Bartosikova L Necas J : Epigallocatechin gallate: a review. *Vet. Med.* 2018;63:443–467. 10.17221/31/2018-VETMED

[24] Grzesik M Naparło K Bartosz G : Antioxidant properties of catechins: Comparison with other antioxidants. *Food Chem.* 2018;241:480–492. 10.1016/j.foodchem.2017.08.117 28958556

[25] Silvestre MA Yániz JL Peña FJ : Role of Antioxidants in Cooled Liquid Storage of Mammal Spermatozoa. *Antioxidants (Basel).* 2021;10:1096. 10.3390/antiox10071096 34356329 PMC8301105

[26] He L He T Farrar S : Antioxidants Maintain Cellular Redox Homeostasis by Elimination of Reactive Oxygen Species. *Cell. Physiol. Biochem.* 2017;44:532–553. 10.1159/000485089 29145191

[27] Nandi A Yan LJ Jana CK : Role of Catalase in Oxidative Stress- and Age-Associated Degenerative Diseases. *Oxidative Med. Cell. Longev.* 2019;2019:9613090. 10.1155/2019/9613090 PMC6885225 31827713

[28] Shafiei M Forouzanfar M Hosseini SM : The effect of superoxide dismutase mimetic and catalase on the quality of postthawed goat semen. *Theriogenology.* 2015;83:1321–1327. 10.1016/j.theriogenology.2015.01.018 25698161

[29] Ranjan R Singh P Gangwar C : Fortification of catalase improves post thaw fertility of goat semen, Iran. *J. Appl. Anim. Sci.* 2021;11:587–593.

[30] Gungor SS Ata A Inac ME : Effects of trehalose and catalase on the viability and kinetic parameters of cryopre-served ram semen. *Acta Sci. Vet.* 2018;46:1–7.

[31] Rubio-Riquelme N Huerta-Retamal N Gómez-Torres MJ : Catalase as a Molecular Target for Male Infertility Diagnosis and Monitoring: An Overview. *Antioxidants (Basel).* 2020;9:78. 10.3390/antiox9010078 31963256 PMC7022443

[32] Mohammadzadeh M Ramazani V Khalili MA : Medium containing different concentrations of catalase as a strategy for optimising sperm parameters and chromatin in normospermic persons. *Andrologia.* 2019;51:e13231. 10.1111/and.13231 30746730

[33] Papas M Arroyo L Bassols A : Activities of antioxidant seminal plasma enzymes (SOD, CAT, GPX and GSR) are higher in jackasses than in stallions and are correlated with sperm motility in jackasses. *Theriogenology.* 2019;140:180–187. 10.1016/j.theriogenology.2019.08.032 31479834

[34] Papas M Catalán J Fernandez-Fuertes B : Specific Activity of Superoxide Dismutase in Stallion Seminal Plasma Is Related to Sperm Cryotolerance. *Antioxidants (Basel).* 2019;8:539. 10.3390/antiox8110539 31717586 PMC6912747

[35] Anggraini T Wilma S Syukri D : Total Phenolic, Anthocyanin, Catechins, DPPH Radical Scavenging Activity, and Toxicity of *Lepisanthes alata (Blume) Leenh.* *Int. J. Food Sci.* 2019;2019:9703176. 31275958 10.1155/2019/9703176 PMC6582855

[36] Kumara P Sunil K Kumar BA : Determination of DPPH free radical scavenging activity by RP-HPLC, rapid sensitive method for the screening of berry fruit juice freeze dried extract. *Nat. Prod. Chem. Res.* 2018;6:341.

[37] Shojaee MS Moeenfard M Farhoosh R : Kinetics and stoichiometry of gallic acid and methyl gallate in scavenging DPPH radical as affected by the reaction solvent. *Sci. Rep.* 2022;12:8765. 10.1038/s41598-022-12803-3 35610331 PMC9130500

[38] Takeshima T Kuroda S Yumura Y : Reactive oxygen species and sperm cells. *Reactive oxygen species (Ros.) in living cells.* London: IntechOpen;2018; (pp.48–73).

[39] Wen F Li Y Feng T : Grape Seed Procyanidin Extract (GSPE) Improves Goat Sperm Quality When Preserved at 4 °C. *Animals (Basel).* 2019;9:810. 10.3390/ani9100810 31618989 PMC6827076

[40] Peris-Frau P Soler AJ Iniesta-Cuerda M : Sperm Cryodamage in Ruminants: Understanding the Molecular Changes Induced by the Cryopreservation Process to Optimize Sperm Quality. *Int. J. Mol. Sci.* 2020;21:2781. 10.3390/ijms21082781 32316334 PMC7215299

[41] Lv C Wu G Hong Q : Spermatozoa Cryopreservation: State of Art and Future in Small Ruminants. *Biopreserv. Biobank.* 2019;17:171–182. 10.1089/bio.2018.0113 30499684

[42] Ayala A Muñoz MF Argüelles S : Lipid peroxidation: production, metabolism, and signaling mechanisms of malondialdehyde and 4-hydroxy-2-nonenal. *Oxidative Med. Cell. Longev.* 2014;2014:360438. 10.1155/2014/360438 PMC4066722 24999379

[43] Alahmar AT : Role of Oxidative Stress in Male Infertility: An Updated Review. *J. Hum. Reprod. Sci.* 2019;12(1):4–18. 10.4103/jhrs.JHRS_150_18 31007461 PMC6472207

[44] Pereira R Sá R Barros A : Major regulatory mechanisms involved in sperm motility. *Asian J. Androl.* 2017;19:5–14. 10.4103/1008-682X.167716 26680031 PMC5227674

[45] Khan IM Cao Z Liu H : Cryopreservation on Spermatozoa Freeze-Thawed Traits and Relevance OMICS to Assess Sperm Cryo-Tolerance in Farm Animals. *Front. Vet. Sci.* 2021;8:609180. 10.3389/fvets.2021.609180 33718466 PMC7947673

[46] Gallo A Esposito MC Tosti E : Sperm Motility, Oxidative Status, and Mitochondrial Activity: Exploring Correlation in Different Species. *Antioxidants (Basel).* 2021;10:1131. 10.3390/antiox10071131 34356364 PMC8301117

[47] Alyethodi RR Sirohi AS Karthik S : Role of seminal MDA, ROS, and antioxidants in cryopreservation and their kinetics under the influence of ejaculatory abstinence in bovine semen. *Cryobiology.* 2021;98:187–193. 10.1016/j.cryobiol.2020.11.002 33476643

[48] Dutta S Majzoub A Agarwal A : Oxidative stress and sperm function: A systematic review on evaluation and management. *Arab. J. Urol.* 2019;17:87–97. 10.1080/2090598X.2019.1599624 31285919 PMC6600059

[49] Ighodaro OM Akinloye OA : First line defence antioxidants-superoxide dismutase (SOD), catalase (CAT) and glutathione peroxidase (GPX): their fundamental role in the entire antioxidant defence grid. *Alex. J. Med.* 2018;54:287–293.

[50] De Luca MN Colone M Gambioli R : Oxidative Stress and Male Fertility: Role of Antioxidants and Inositols. *Antioxidants (Basel).* 2021;10:1283. 10.3390/antiox10081283 34439531 PMC8389261

[51] Chen M Liu W Li Z : Effect of epigallocatechin-3-gallate (EGCG) on embryos inseminated with oxidative stress-induced DNA damage sperm. *Syst. Biol. Reprod. Med.* 2020;66:244–254. 10.1080/19396368.2020.1756525 32427532

[52] Hill EK Li J : Current and future prospects for nanotechnology in animal production. *J. Anim. Sci. Biotechnol.* 2017;8:26. 10.1186/s40104-017-0157-5 28316783 PMC5351054

[53] Daramola JO Adekunle EO Oke OE : Effects of pyridoxine in combination with different antioxidants on viability and oxidative stress parameters of cryopreserved goat buck semen. *Arch. Zootec.* 2017;66:15–21. 10.21071/az.v66i253.2120

[54] Susilowati S Sardjito T Mustofa I : Effect of green tea extract in extender of Simmental bull semen on pregnancy rate of recipients. *Anim. Biosci.* 2021;34:198–204. 10.5713/ajas.20.0025 32299169 PMC7876723

[55] Mustofa I Susilowati S Suprayogi TW : Epigallocatechin-3-gallate chitosan nanoparticles in an extender improve the antioxidant capacity and post-thawed quality of Kacang Goat semen. *BioStudies.* 2022;S-BSST924. Reference Source 10.12688/f1000research.127744.3 PMC12521894 41103504

[56] Mustofa I Susilowati S : Author Checklist - Full_EGCG CNPs.pdf. figshare. Poster. 2022. 10.6084/m9.figshare.21531213.v1

