# Supplementary material for: Epigallocatechin-3-gallate chitosan nanoparticles in an extender improve the antioxidant capacity and post-thawed quality of Kacang goat semen
Source: F1000Res. 2025 Sep 5;12:32. Originally published 2023 Jan 9. [Version 3] doi: 10.12688/f1000research.127744.3 (PMC12521894; doi:10.12688/f1000research.127744.3)
Supplement: Peer review history

# Reviewer report: Reviewer response for version 3

**DOI:** 10.5256/f1000research.187380.r416962

**Published:** 2025-10-14

**Authors:** Sajeda Mahdi Eidan

**Affiliations:** 1 University of Baghdad, Baghdad, Iraq

1. The terminology should be standardized throughout manuscript according to the first abbreviation. For instance, MPI should be used consistently instead of IPM, EGCG CNPs should replace with EGCG chitosan nanoparticles (CNPs), and viability should replace with living cells.
2. Don’t use brackets next to each other “(T0) (p < 0.05) (Table 3) “, combine them to one bracket & separate the references with semicolons “(T0;p < 0.05; Table 3)”
3.  The equilibrium period  for glycerol during the cooling stage is one hrs. which is a short duration. Please justify this with an appropriate reference or clarify the basis for this statement. Kindly check 
4. The correct reference for plasma membrane integrity is Jeyendran et al., (1984). Please confirm this reference ‘ Jeyendran, R,S., H.H. Vander van, M. Pelaez, B.G. Crabo and  L.J.D.1984. Development of an assay to assess the functional integrity of the human sperm membrane and its relationship other semen characteristics. J. Reprod. Fertil.,70:219-228.’’
5. Kindly check the MDA statistical analysis (Table 3), as it seems that no significant differences exist among treatments.
6. “Semen that was frozen without antioxidant EGCG CNPs in the extender (T0 group) exhibited lowest sperm MPI, living cells, and motility both in the pre-freezing and post-thawed conditions (p < 0.05). Adding EGCG CNPs at1.5and2.0 μg/ mL to the SM–EY extender improved semen quality compared with the control group(T0)(p<0.05),with no significant difference between the two doses (Table 4).” Referring to Table 4, no significant differences are observed among treatments. All means are marked with ‘a’ except T3&T4 which have both a & b. Please verify and correct any inconsistencies in the statistical analysis and notation.

To provide more comprehensive evaluation of the treatments effects, it would be beneficial to include data on fertility outcomes of goats inseminated with treated semen, especially this study involves an endangered goat breed (Kacang goats) as stated in introduction.

1. Following addition of data on acrosome integrity, sperms abnormalities, sperm DNA damage, and   fertility percentages. The conclusion should be reformulated to reflects these findings

Is the work clearly and accurately presented and does it cite the current literature?

Yes

If applicable, is the statistical analysis and its interpretation appropriate?

Partly

Are all the source data underlying the results available to ensure full reproducibility?

Partly

Is the study design appropriate and is the work technically sound?

Yes

Are the conclusions drawn adequately supported by the results?

Partly

Are sufficient details of methods and analysis provided to allow replication by others?

Partly

Reviewer Expertise:

Semen cryopreservation, metabolic biomarkers, Fertility of males and females, molecular biology related to fertility , Nutrition related to fertility

I confirm that I have read this submission and believe that I have an appropriate level of expertise to confirm that it is of an acceptable scientific standard, however I have significant reservations, as outlined above.

# Reviewer report: Reviewer response for version 3

**DOI:** 10.5256/f1000research.187380.r412691

**Published:** 2025-09-16

**Authors:** Daniel Berean

**Affiliations:** 1 University of Agricultural Sciences and Veterinary Medicine Cluj-Napoca, Cluj-Napoca, Romania

Congratulations on your study. Improving sperm cell characteristics after the thawing process is not easy to achieve and requires meticulous and hard work. The importance of such research is continuously increasing, particularly in the small ruminant sector, where sperm conservation techniques are not as well developed as in large ruminants or the equine sector.

 The article is well written and has a logical structure; however, there are some gaps that need to be addressed. I will enumerate them below. Where it is no longer possible to address certain points, I suggest kindly including them in the limitations of the study as a final paragraph in the discussion section.

 I suggest revising the title to make it more concise and readable, for example: "Epigallocatechin-3-gallate Chitosan Nanoparticles Enhance Antioxidant Capacity and Post-Thaw Quality of Kacang Goat Semen."

 In the results section of the abstract, it appears that only this concentration (1.5/ml) improved the parameters. Please clarify this point or specify that 1.5 µg/mL was the optimal concentration.

 In the conclusion of the abstract, the value of 1 µg/mL is already mentioned; I suggest here including the most effective concentration and, in the results section, providing a summary of the effects of the other concentrations.

 Please verify that the introduction section addresses the characteristics and importance of all the sperm parameters investigated. I suggest presenting this information in a chronological and logical order rather than providing an amalgam of details, which forces the reader to discern what is important. Additionally, a brief discussion on sperm evaluation methods and how these parameters are assessed should be included.

 A clearer and simpler description of the dilutions is needed, as the current form does not clearly explain what happened after semen collection. For example, were the samples mixed or kept separate? The dilution rate should also be specified. Additionally, information about the bucks is missing, were they collected prior to estrus, and was a doe in estrus used for sperm collection? The main gap in the Materials and Methods section is the use of classical, non-automated methods for assessing sperm characteristics. This limitation should be explicitly acknowledged and categorized as an important limitation of the study.

 In some parts of the article, the authors refer to motility, while in other parts they mention progressive motility. It should be clarified which parameter was actually assessed, or, if both were measured, the text should be revised to clearly distinguish and describe each.

 In the discussion section, more data from your own study should be included and developed in comparison with other studies. In its current form, the authors refer extensively to literature data while only occasionally discussing their own results.

Is the work clearly and accurately presented and does it cite the current literature?

Partly

If applicable, is the statistical analysis and its interpretation appropriate?

Yes

Are all the source data underlying the results available to ensure full reproducibility?

Partly

Is the study design appropriate and is the work technically sound?

Partly

Are the conclusions drawn adequately supported by the results?

Yes

Are sufficient details of methods and analysis provided to allow replication by others?

Yes

Reviewer Expertise:

Small ruminants reproduction, sperm cryopreservation

I confirm that I have read this submission and believe that I have an appropriate level of expertise to confirm that it is of an acceptable scientific standard, however I have significant reservations, as outlined above.

## References

[1] : Economical implications and the impact of gonadotropin-releasing hormone administration at the time of artificial insemination in cows raised in the extensive system in North Romania. *Frontiers in Veterinary Science* .2023;10: 10.3389/fvets.2023.1167387 10.3389/fvets.2023.1167387 PMC1017249637180075

# Reviewer report: Reviewer response for version 2

**DOI:** 10.5256/f1000research.146355.r318492

**Published:** 2024-09-12

**Authors:** CHETNA GANGWAR

**Affiliations:** 1 ICAR-Central Institute for Research on Goats, Uttar Pradesh, India

1. Authors used different nomenclature at different places like MPI and IPM.

 2. In Preparation of EGCG CNPs - Author used this statement - This solution was centrifuged at 21,952 × *g* for 10 min. How this can accurately done.

 3. In estimation of ***MDA levels -***  author write this - A semen sample (100 μL) and MDA kits (0, 1, 2, 3, 4, 5, 6, 7, and 8 μg/mL of malondialdehyde) were added, again this incorrect.

 4. Moreover, many microscopic evaluation can be done to assess the NPs effect.

 6. In Catalase test- A 30 mM H _2_O _2_ phosphate-buffered solution [1 mL; comprising 0.34 mL of 30% H _2_O _2_ diluted in fresh phosphate buffer (50 mM and pH 7)] was added to a semen sample (2 mL) , whether this sample is diluted or neat. Generally bucks give 0.5 to 1.5 ml semen. If you used whole semen, how you did other parameters.

 5. No complete detail in statistical analysis is included.

 6. There are many mistakes in English.

Is the work clearly and accurately presented and does it cite the current literature?

Partly

If applicable, is the statistical analysis and its interpretation appropriate?

Yes

Are all the source data underlying the results available to ensure full reproducibility?

Partly

Is the study design appropriate and is the work technically sound?

Partly

Are the conclusions drawn adequately supported by the results?

Partly

Are sufficient details of methods and analysis provided to allow replication by others?

Partly

Reviewer Expertise:

Andrology, Buck semen cryopreservation

I confirm that I have read this submission and believe that I have an appropriate level of expertise to state that I do not consider it to be of an acceptable scientific standard, for reasons outlined above.

# Reviewer report: Reviewer response for version 2

**DOI:** 10.5256/f1000research.146355.r168663

**Published:** 2023-04-05

**Authors:** Muhammed Duman

**Affiliations:** 1 Department of Aquatic Animal Diseases, Faculty of Veterinary Medicine, Bursa Uludag University, Bursa, Turkey

The revised version meets all criteria mentioned by reviewers. The authors have done all suggested sections based on reviewer reports.

Is the work clearly and accurately presented and does it cite the current literature?

Yes

If applicable, is the statistical analysis and its interpretation appropriate?

Yes

Are all the source data underlying the results available to ensure full reproducibility?

Yes

Is the study design appropriate and is the work technically sound?

Yes

Are the conclusions drawn adequately supported by the results?

Yes

Are sufficient details of methods and analysis provided to allow replication by others?

Yes

Reviewer Expertise:

Semen quality and additives

I confirm that I have read this submission and believe that I have an appropriate level of expertise to confirm that it is of an acceptable scientific standard.

# Reviewer report: Reviewer response for version 1

**DOI:** 10.5256/f1000research.140281.r160298

**Published:** 2023-02-17

**Authors:** Maria Madalena Pessoa Guerra

**Affiliations:** 1 mmpguerra@gmail.com, University Federal Rural of Pernambuco (UFRPE), Recife, Brazil

The article aims to determine the addition of epigallocatechin-3-gallate chitosan nanoparticles (EGCG CNPs) to skim milk–egg yolk (SM–EY) extender to obtain the best possible quality of post-thawed Kacang buck semen for AI. The work is well written and can make a great contribution to the freezing of goat semen. However, you need to make a lot of corrections before it's indexed.

1. The term "viability" is not appropriate, as it does not define well what was evaluated in the spermatic cell. I suggest replacing it with "living cells"
2. With the exception of IPM, the addition of EGCG CNPs at doses of 1.5 and 2.0 μg/mL extended post-thawed catalase, DPPH, viability, and sperm motility and decreased MDA levels (p < 0.05) than those of control group.
3. The conclusion of the abstract is not consistent with the results, as 1.5 μg/mL and 2.0 μg/mL of EGCG CNPs in this extender increased the antioxidant capacity and post-thawed quality of Kacang buck semen.
4. What is the methodology used to cool semen from room temperature (25°C) to 5°C for 1 hour?
5. Results: Part of the first paragraph can transfer to the discussion.
6. This statement “The addition of EGCG CNPs in extender resulted in higher catalase and DPPH levels and lower MDA levels (p < 0.05) in optimum dose of 1.5 μg/mL of extender (T3) than those in the T0 group (p < 0.05) (Table 3). “ is not correct, because the significant difference occurred at doses 1.5 and 2.0 μg/mL.
7. This statement “The addition of EGCG CNPs at 1.5 μg/mL of extender (T3) was optimum dose for increasing of sperm viability, motility and IPM (p < 0.05) than those in the T0 group (Table 4).” also does not agree with the results, since there was a difference between analyses: MPI was better from T4 (2.0 μg/mL), while live and motility were better with T3 (1.5 μg/mL), and T4 (2.0 μg/mL).
8. The discussion needs to be modified. Little of the results obtained were discussed with the literature data. Looks like a literature review.
9. This statement “The lower MDA levels in post-thawed semen of T3 group indicated the decreasing of oxidative stress, followed by an increase in membrane fluidity and a lower percentage of acrosomal damage owing to the rearrangement of membrane lipids and proteins.”  should be modified, because groups T2, T3 and T4 did not differ from each other and were inferior to the T) (control).
10. The statement that "A higher value of DPPH in the T3 than those of T0 group represents more effective free radical scavenging." should also be modified, since T4 did not differ from T3.
11. The statement that “Adding EGCG CNPs at 1.5 μg/mL of SM–EY extender improved semen quality compared with that of the control group.” should be modified, as T3 and T4 did not differ from each other.
12. The statement of the conclusion that “Indeed, the addition of 1.5 μg/mL EGCG CNPs to the SM–EY extender increased the antioxidant capacity and quality of Kacang buck post-thawed semen.” must be modified.
13. The title of the tables should explain the analyses performed, in the same order in which the results are presented below.

Is the work clearly and accurately presented and does it cite the current literature?

Yes

If applicable, is the statistical analysis and its interpretation appropriate?

Yes

Are all the source data underlying the results available to ensure full reproducibility?

Yes

Is the study design appropriate and is the work technically sound?

Yes

Are the conclusions drawn adequately supported by the results?

No

Are sufficient details of methods and analysis provided to allow replication by others?

Yes

Reviewer Expertise:

-

I confirm that I have read this submission and believe that I have an appropriate level of expertise to state that I do not consider it to be of an acceptable scientific standard, for reasons outlined above.

# Response

**Published:** 2023-03-30

**Authors:** Imam Mustofa

**Affiliations:** Airlangga University, Indonesia

Thank you the suggestions for improvement of our article from the reviewers. We have corrected this article as follows. We have revised the narration in the Abstract, Introduction, Results, Discussion, and Conclusion based on data and suggestion of reviewers.

# Reviewer report: Reviewer response for version 1

**DOI:** 10.5256/f1000research.140281.r161557

**Published:** 2023-02-07

**Authors:** Muhammed Duman

**Affiliations:** 1 Department of Aquatic Animal Diseases, Faculty of Veterinary Medicine, Bursa Uludag University, Bursa, Turkey

I would like to suggest some additions to the manuscript, including the introduction and discussion sections. When I read the article, it sounds much more scientific and understandable. I do not see any methodological error, but I think additions for improving post-thawed semen quality in goats are popular subjects. In this updated area, the authors need to add some updated additives to improve semen quality in the introduction section and need to discuss them in the discussion. Why Epigallocatechin-3-gallate chitosan nanoparticles are well comparing other antioxidants and freezer protectors in goat semen. 

 I am suggesting some minor points below:

- Introduction should be enlarged
- There are some other studies about additions increase post-thawed semen quality such as fish seminal plasma, egg yolk, curcumin, inositol and carnitine. Authors should mention other articles in the introduction section some samples given below
  Information about post-thawed semen quality in goats is very critical subject and authors need to mention what is the updated researches on this subject. For example:
  - https://doi.org/10.1016/j.anireprosci.2015.11.017
  - https://doi.org/10.1016/j.smallrumres.2009.11.015
  - http://zsp.com.pk/pdf47/119-124%20(16)%20PJZ-1915-14%2011-12-14%20page%20proof%20PJZ.pdf
  - http://www.pvj.com.pk/pdf-files/34_3/347-350.pdf
  - https://doi.org/10.1111/and.13555
- The mentioned studies in introduction section, should be discussed here
- The reference (56) is not relevant to the study and should be discarded

After revisions, the manuscript will be high impact and mostly cited because of comprising an updated subject.

Is the work clearly and accurately presented and does it cite the current literature?

Yes

If applicable, is the statistical analysis and its interpretation appropriate?

Yes

Are all the source data underlying the results available to ensure full reproducibility?

Yes

Is the study design appropriate and is the work technically sound?

Yes

Are the conclusions drawn adequately supported by the results?

Yes

Are sufficient details of methods and analysis provided to allow replication by others?

Yes

Reviewer Expertise:

Semen quality and additives

I confirm that I have read this submission and believe that I have an appropriate level of expertise to confirm that it is of an acceptable scientific standard, however I have significant reservations, as outlined above.

## References

[1] : Effect of rainbow trout (Oncorhynchus mykiss) seminal plasma on the post-thaw quality of ram semen cryopreserved in a soybean lecithin-based or egg yolk-based extender. *Anim Reprod Sci* .2016;164: 10.1016/j.anireprosci.2015.11.017 97-104 10.1016/j.anireprosci.2015.11.017 26685096

[2] : The effect of antioxidants on post-thawed Angora goat (Capra hircus ancryrensis) sperm parameters, lipid peroxidation and antioxidant activities. *Small Ruminant Research* .2010;89(1) : 10.1016/j.smallrumres.2009.11.015 24-30 10.1016/j.smallrumres.2009.11.015

[3] : Effect of butylated hydroxytoluene on post-thawed semen quality of beetal goat buck, Capra hircus. *Pakistan Journal of Zoology* .2015;47:119-24 Reference source

[4] : Comparison of cryopreservative effect of different levels of omega-3 egg-yolk in citrate extender on the quality of goat spermatozoa. *Pakistan Veterinary Journal* .2014;34(3) :347-350 Reference source

[5] : Goat semen cryopreservation with rainbow trout seminal plasma supplemented lecithin-based extenders. *Andrologia* .2020;52(4) : 10.1111/and.13555 e13555 10.1111/and.13555 32107791

# Response

**Published:** 2023-03-30

**Authors:** Imam Mustofa

**Affiliations:** Airlangga University, Indonesia

Thank you the suggestions for improvement of our article from the reviewers. We have corrected this article as follows. We have added some other studies about additions substances in extender to increase post-thawed goats semen quality in the Introduction and Discussion section. The relevant references were added.
